# Sport-Related Portal Vein Thrombosis: An Unusual Complication

**DOI:** 10.1155/2017/9324246

**Published:** 2017-11-12

**Authors:** Igor Dumic, Nikola Tankosic, Milica Stojkovic Lalosevic, Tamara Alempijevic

**Affiliations:** ^1^Department of Hospital Medicine, Mayo Clinic Health System, Eau Claire, WI, USA; ^2^Mayo Clinic College of Medicine and Science, Rochester, MN, USA; ^3^North Bronx Health Network, North Central Bronx Hospital, Bronx, NY, USA; ^4^Icahn School of Medicine at Mount Sinai, New York, NY, USA; ^5^Department of Gastroenterology and Hepatology, Clinical Center of Serbia, Belgrade, Serbia; ^6^School of Medicine, University of Belgrade, Belgrade, Serbia

## Abstract

Portal vein thrombosis (PVT) is an uncommon condition usually associated with hypercoagulable states or liver cirrhosis. PVT due to sports-related injuries is rarely reported and, to the best of our knowledge, only two cases have been reported thus far. Brazilian jiu-jitsu (BJJ) is a form of martial arts and is considered very safe with minimal risk for injury. It has growing popularity worldwide. Here, we report the first case of PVT secondary to abdominal trauma related to the practice of (BJJ) in an otherwise healthy 32-year-old man with no other traditional risk factors for PVT.

## 1. Introduction

PVT is a rare condition in otherwise healthy individuals. It is most commonly associated with liver cirrhosis, malignancy, Philadelphia chromosome-negative chronic myeloproliferative neoplasms (MPN), and congenital or acquired prothrombotic disorders. Abdominal trauma is an uncommon cause of PVT. BJJ is a martial art, combat sport that focuses on grappling and ground fighting. It is considered to be one of the safest sports in the category; it is rarely associated with severe injuries and is one of the fastest growing sports in the world. Here, we report a case of previously healthy 32-year-old man who developed abdominal pain 2 weeks after starting BJJ and was diagnosed with PVT. To the best of our knowledge, there are only two case reports of sport-related PVT published thus far and this is the first case report of PVT caused by injuries from BJJ.

## 2. Case Report

A 32-year-old, previously healthy man presented to the emergency department with complaint of 3 weeks of gradually worsening abdominal pain. His pain started approximately 2 weeks after he had started practicing BJJ. He initially attributed the pain to BJJ-related contusion caused by the sport technique which included prolonged compression on abdomen by opponent's knee, which is known as “knee on stomach technique.” He reduced the frequency of his training sessions; however, the pain persisted and worsened. It was periumbilical and constant, ranging in severity from 5 to 7 out of 10. Lying in bed would help relieve the pain and movements would intermittently exacerbate it. Patient complained of some nausea without vomiting but denied any fevers, chills, or recent travel. Frequency of bowel movements was reduced but stool was normal in color and consistency without blood or mucus. He took no medications, supplements, or steroids but tried some antacids and Pepto-Bismol for abdominal pain without relief. He was a former smoker and used alcohol socially but denied any illicit drug use. He was married and monogamous. Patient also denied any abdominal surgeries in the past or any family history of liver disease or venous thromboembolism.

On presentation, he was afebrile with normal vital signs and appeared in no distress. Physical exam was unremarkable with the exception of periumbilical and right upper quadrant abdominal pain on deep palpation. There was no rebound tenderness, hepatosplenomegaly, icterus, telangiectasia, or flapping tremors. Bowel sounds were normal.

Routine laboratory testing revealed largely normal values with an exception of mildly elevated liver enzymes: AST was 77 U/L and ALT was 44 U/L. These included hemoglobin of 15 g/dL, white cell count of 10.3 × 10^9^/L with normal differential platelet count of 179 × 10^9^/L, and normal total bilirubin, alkaline phosphatase, amylase, lipase, lactic acid, electrolytes, and renal function tests as well as PTT, INR, fibrinogen level, and thrombin time. Extensive prothrombotic work-up was initiated and included protein C, protein S, antithrombin functional activity, anti-nuclear antibodies, lupus anticoagulant, anti-*β*2 glycoprotein antibodies, anticardiolipin antibodies, JAK2 V617F, and Factor V Leiden mutation as well as PNH flow cytometry for CD 56 and CD 59 which were all negative. Testing for CALR exon 9 mutation was unavailable at our institution. Homocysteine and ferritin level were normal. There was no evidence of hepatitis A, B, or C infection. Anti-smooth muscle antibodies and antimitochondrial antibodies for autoimmune liver disease were negative as well.

**Figure 1:**
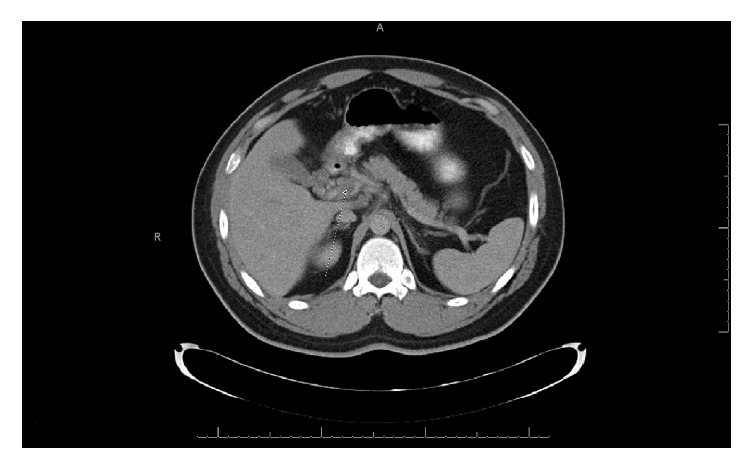
Partially occluded main portal vein (arrow).

Abdominal CT scan with and without IV and oral contrast showed partial thrombosis of the main portal vein extending to the left branch (Figure 1). Partial thrombosis of superior mesenteric vein (SMV) with marked distention and extensive surrounding infiltrative changes were seen as well. There were collaterals noted at the level of the porta hepatis and significant edema of proximal jejunum. There was no evidence of pneumatosis, ascites, or intra- or extrahepatic masses.

**Figure 2:**
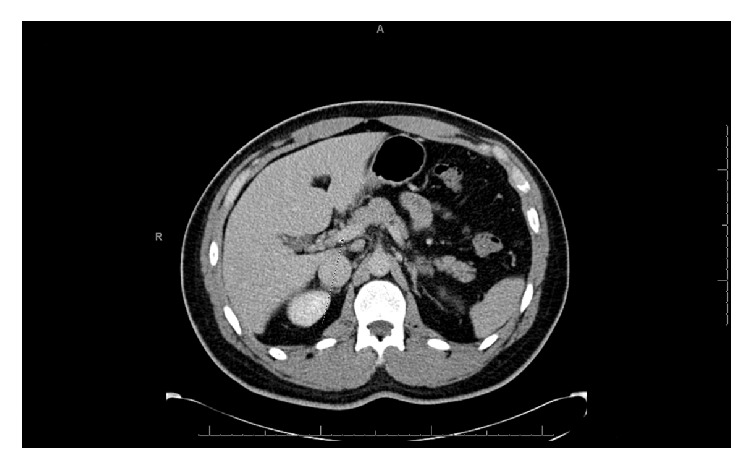
Resolution of previously occluded main portal vein following 6 months of anticoagulation therapy (arrow).

The patient was started on anticoagulation with warfarin and was bridged for 4 days with low-molecular-weight heparin until his INR became therapeutic. His abdominal pain resolved on hospital day 3 and he was gradually started on a diet which he tolerated well. He was discharged home to complete 6 months of anticoagulation with warfarin with goal INR between 2 and 3. After 6 months of anticoagulation therapy, follow-up CT scan of abdomen was performed, which showed complete resolution of previously occluded portal vein (Figure 2). SMV thrombosis and jejunal edema were completely resolved as well. Since all hypercoagulability work-up came back negative, the abdominal trauma related to sport was the only clearly identifiable risk factor in this patient.

## 3. Discussion 

Portal vein thrombosis is defined by development of thrombus in the portal vein itself or one of its branches [1]. Lifetime risk of developing PVT in general population is found to be 1% [2, 3]. Acute PVT is considered when symptoms develop <60 days before presentation. In chronic PVT, there is a formation of numerous collateral veins around the thrombosed portal vein known as portal cavernomas [4]. Patients with cirrhosis have an incidence of PVT between 0.6% and 16%. In patients with more advanced disease and especially in those with hepatocellular carcinoma, incidence goes up to 35% [4, 5]. Our patient, interestingly, presented within 60 days from symptoms onset; however, he was found to have portal cavernomas, suggesting that the process was more subacute. Indeed, there is evidence that these collateral blood vessels start forming within first few days following thrombus formation [6, 7].

Brazilian jiu-jitsu is a form of martial arts with rapidly growing popularity in the United States as well as internationally. BJJ maneuvers allow a physically weaker person to successfully subdue stronger opponents by using proper techniques. One of the techniques is called “knee on stomach” which involves prolonged abdominal compression by opponent's knee. This was a technique that our patient was trying to master. Most common reported injuries related to BJJ are orthopedic injuries with elbow being most commonly injured [8].

PVT most commonly occurs in patients with liver cirrhosis, intraabdominal malignancy (especially hepatocellular carcinoma), intraabdominal infection (pylephlebitis and liver abscess), inherited or acquired prothrombotic disorders, MPN, or autoimmune diseases and rarely in nephrotic syndrome. Sport-related trauma leading to PVT is rare with only two cases reported in literature, one in scuba diver and another in rugby player [9, 10]. The proposed mechanisms by which these sports-related injuries cause PVT are most likely related to local endothelial damage to veins of abdominal wall and periportal hematoma formation with subsequent stasis in portal vein leading to thrombus formation [11, 12]. Alternatively, more forceful abdominal trauma might shear the portal vein and induce endothelial damage that will serve as starting point for thrombus formation and its subsequent propagation [13].

In the current case, given the close temporal relation between beginning of practicing BJJ and symptom onset and by ruling out other known etiologies of PVT, we are pretty confident that this was trauma-related PVT. Noncirrhotic, nonmalignant PVT is usually caused by combination of local and systemic risk factors. Systemic risk factors are more common and, among them, either overt or occult MPN are most commonly the cause [14]. Despite extensive work-up, the etiology remains elusive in about 25% of cases [15, 16]. Among these 25%, so-called idiopathic cases, we speculate that some might be caused by yet unknown prothrombotic disorder. In the setting of portal hypertension and splenomegaly, evidence of myeloproliferation might not be always evident on peripheral blood testing. JAK2 V617F mutation is the most important thrombophilic risk factor and has been described in up to 60% of patients with PVT [17]. Recently, CALR gene exon 9 mutation has been described in majority of patients who have JAK2 nonmutated MPN [18]. While we could not perform testing for CALR mutation in our patient, we consider occult MPN to be unlikely cause in this case, since patient remained symptoms- and disease-free 2 years following the initial diagnosis of PVT.

Symptoms of PVT vary from nonspecific abdominal pain to life-threatening intestinal ischemia in acute cases, where there is no time for collaterals to form and thrombus propagates into SMV. In chronic PVT, most common presentation is hematemesis from ruptured esophageal varices or bleeding from portal hypertensive gastropathy [4, 6]. Lately, increased incidence of confirmed PVT diagnoses is likely secondary to widespread use of abdominal imaging, particularly color Doppler ultrasonography, computed tomography, and magnetic resonance imaging. Ultrasound with color Doppler imaging has 98% negative predictive value and is preferred imaging modality [4]. Advantage of using CT scan is that it can at the same time easily demonstrate malignant growth causing PVT; however, it has to be used with IV contrast, which requires extra caution in patients with renal insufficiency, which is common in advanced liver disease.

Currently, there are no clear guidelines regarding optimal treatment for PVT. Clinical data supporting anticoagulation treatment are based on case reports, expert opinions, and retrospective and prospective studies. Randomized trials are difficult to conduct due to rarity and heterogeneity of this disorder. Anticoagulation appears to be safe for acute cases of PVT, especially in noncirrhotic, nonmalignant PVT [16, 19]. Patients who have splenic vein thrombosis associated with PVT and ascites have lower chance for recanalization, so other treatment options should be considered [19]. Patients with chronic PVT are in more difficult situation, since most of them have esophageal varices prone to bleeding in the setting of anticoagulation; however, mortality in that setting has been shown to be relatively low, 1.5% [16]. There are case reports of using thrombolytic therapy via transjugular or percutaneous transhepatic approach for treatment of patients with acute PVT. However, these procedures are invasive and are poorly evaluated and should be considered only for patients who failed traditional approach and have low chance of recanalization of portal flow despite anticoagulation [16, 20, 21]. Our patient received anticoagulation with warfarin with goal INR between 2 and 3. On repeated CT scan following completion of anticoagulation, PVT and SMV thrombosis were resolved as well as edema surrounding jejunum. He remained without any recurrence of thrombosis during 2-year follow up. Prognosis of PVT mostly depends on underlying cause and condition in which thrombosis occurred. Survival of patients with PVT in the setting of cirrhosis can be estimated using MELD score. Majority of patients with noncirrhotic, nonmalignant PVT have better prognosis compared to patients with cirrhosis-associated PVT and PVT associated with advanced malignancy or rapidly progressing myeloproliferative disorder. In those cases, survival is limited by underlying disease rather than PVT itself. One-year survival varies from 80% to 95% and 3-year survival from 75% to 90% in patient with chronic PVT [22].

## 4. Conclusion

This is the first case report that describes connection between practicing Brazilian jiu-jitsu and development of portal vein thrombosis. BJJ is becoming popular sport among young, healthy individuals. Albeit rare, blunt abdominal trauma is a well-recognized risk factor for development of PVT; therefore, we believe that it is important to consider PVT in differential diagnosis of abdominal pain in a healthy person practicing BJJ or other contact sports associated with repetitive abdominal traumas.
